# Oxidant-Dependent Switch of a Molybdenum(VI) Tetrazolate Complex from a Homogeneous to a Self-Separating Catalyst for Olefin Epoxidation

**DOI:** 10.1021/acs.iecr.5c01997

**Published:** 2025-08-18

**Authors:** Martinique S. Nunes, Diana M. Gomes, Patrícia Neves, Ana C. Gomes, Ricardo F. Mendes, Filipe A. Almeida Paz, Isabel S. Gonçalves, Anabela A. Valente, Martyn Pillinger

**Affiliations:** CICECO − Aveiro Institute of Materials, Department of Chemistry, 56062 University of Aveiro, Campus Universitário de Santiago, Aveiro 3810-193, Portugal

## Abstract

Although several decades have passed since the introduction of homogeneous molybdenum catalysts for the bulk industrial production of epoxides from light olefins, the development of recyclable catalytic systems to produce more complex epoxides remains a challenge. In this work, we present a strategy for preparing a self-separating catalyst by exploiting reaction-induced precipitation, starting from the molybdenum­(VI) tetrazolate complex [MoO­(O_2_)­(pto)_2_] (Hpto = 5-(2-pyridyl-1-oxide)­tetrazole), which was synthesized via a one-pot approach and crystallographically characterized. High epoxide selectivities (96–100%) were achieved at high conversions (88–100%) under mild conditions (70 °C) in all the studied reactions, from that of the model substrate *cis*-cyclooctene to the epoxidation of biobased dl-limonene and fatty acid methyl esters. The catalytic reaction is homogeneous using *tert*-butyl hydroperoxide as the oxidant, whereas with hydrogen peroxide, a transformation to a self-separating catalyst takes place, which combines the high catalytic activity of a homogeneous catalyst with the easy recovery and reuse of a heterogeneous catalyst.

## Graphical abstract

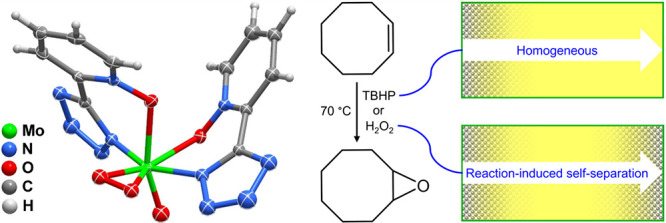

## 1 Introduction

Metal carbonyl compounds have long held an important position in industrial catalysis for organic synthesis.^1^ For example, in the Monsanto and Cativa processes, rhodium and iridium carbonyl complexes are used as catalysts to produce acetic acid via the carbonylation of methanol.^2^ Molybdenum hexacarbonyl, Mo­(CO)_6_, has been used in various organic transformations such as the Pauson–Khand reaction, carbonyl insertion, olefin epoxidation, and rearrangement reactions.^3^ In the Halcon–ARCO–Lyondell hydroperoxide process for the epoxidation of propylene, the precatalyst Mo­(CO)_6_ is oxidized in situ to catalytically active oxo-peroxo species.^4,5^ Owing to its commercial availability, relatively low cost, and high stability toward air and moisture, Mo­(CO)_6_ is attractive to be used either directly as a (pre)­catalyst^3^ or indirectly as a precursor for the synthesis of molybdenum carbonyl complexes^5^ or simply as a solid source of CO in carbonylation reactions.^6^

When Mo­(CO)_6_ is used as an organometallic precursor, the most common implementation is its reaction with an organic ligand (L) to give a heteroleptic complex of the type [Mo­(CO)_*x*_(L)_*y*_]. Like Mo­(CO)_6_, these complexes can be used as precatalysts for olefin epoxidation, with the difference being that the ligand L (typically an N-donor ligand) can exert a structure-directing influence on the outcome of oxidative decarbonylation (OD),^5^ which has resulted in the isolation of diverse Mo^VI^ compounds such as mononuclear [MoO­(O_2_)_2_(bis­(pyrazolyl)­methane)],^7^ dinuclear [{MoO_2_(tris­(3,5-dimethyl-1-pyrazolyl)­methane)}_2_(μ_2_-O)]^2+^,^8^ tetranuclear [Mo_4_O_12_(pzpy)_4_] (pzpy = 2-[3(5)-pyrazolyl]­pyridine),^9^ octanuclear [Mo_8_O_24_(4,4′-di-*tert*-butyl-2,2′-bipyridine)_4_],^10^ and polymeric [MoO_3_(2,2′-bipyridine)].^10^ The behavior of these compounds as homogeneous or heterogeneous catalysts or as reservoirs of active species that break off and dissolve is dependent on the ligand types, molecular and/or solid-state structures, and conditions used for catalysis.

The tetranuclear complex [Mo_4_O_12_(pzpy)_4_] was obtained upon the oxidation of [Mo­(CO)_4_(pzpy)] with *tert*-butyl hydroperoxide (TBHP).^9^ Azolylpyridines like pzpy are attractive multitopic ligands owing to the presence of basic N atoms and acidic N–H groups, which opens the door to multiple coordination modes, especially if the acidic proton is removed to give anionic moieties like pyrazolate, triazolate, or tetrazolate groups.^11–22^ Whereas many Mo­(VI) coordination compounds are known with pyrazolylpyridine ligands, ranging from discrete molecules to extended structures,^9,23–31^ only two compounds have been reported containing tetrazolylpyridines, namely, the dioxomolybdenum­(VI) complexes (H_2_pytz)­[MoO_2_Cl_2_(pytz)] (Hpytz = 5-(2-pyridyl)­tetrazole)^32^ and [MoO_2_Cl_2_(*t*Bu-pytz)] (*t*Bu-pytz = 2-*tert*-butyl-5-(2-pyridyl)–2*H*-tetrazole).^33^ A related complex, [MoO_2_Cl_2_(Hpto)], containing the pyridine N-oxide derivative of Hpytz (Hpto = 5-(2-pyridyl-1-oxide)­tetrazole) was also recently reported.^34^ In the epoxidation of *cis*-cyclooctene (Cy), the complexes (H_2_pytz)­[MoO_2_Cl_2_(pytz)] and [MoO_2_Cl_2_(Hpto)] led to homogeneously catalyzed reactions with very high activity (100% epoxide yield within 1 h).

Despite the success of using complexes of the type [MoO_2_Cl_2_(L)] as olefin epoxidation catalysts, the presence of Cl ligands is a potential drawback as these are released upon hydrolysis or ligand exchange, leading to negative effects on the reaction efficiency (by causing, for example, selectivity problems, product contamination, and/or catalyst deactivation) as well as equipment corrosion and generation of halide-containing effluents. These issues are avoided by using complexes of the type [Mo­(CO)_*x*_(L)_*y*_] as catalyst precursors, which release only easily separated gaseous CO/CO_2_ as coproducts of the catalyst formation step, avoiding product contamination (although effluent gas treatment processes would need to address the potential hazards associated with CO, including its toxicity and flammability). To date, catalytic studies involving heteroleptic precursors of the type [Mo­(CO)_*x*_(azolylpyridine)_*y*_] have been restricted to olefin epoxidation and sulfide oxidation with oxomolybdenum catalysts obtained from tri/tetra-carbonyl complexes containing pyrazolylpyridine ligands.^9,35,36^ This fact, together with the promising catalytic results obtained for the pytz/Hpto dichlorodioxomolybdenum­(VI) complexes, motivated the present study in which the oxo-peroxo complex [MoO­(O_2_)­(pto)_2_] (**1**) was obtained in a one-pot manner starting from Mo­(CO)_6_ and Hpto. The catalytic performance of **1** for olefin epoxidation was explored using different oxidants, solvents, and substrates, from Cy as a model substrate to biomass-derived olefins. Reaction-induced self-separating (RISS) behavior was observed when using H_2_O_2_ as an oxidant, combining the best features of homogeneous (active species that are easily accessible to bulky substrates, favoring the reaction kinetics) and heterogeneous (facilitated separation of the solid catalyst) catalysis.

## 2 Experimental Section

### 2.1 Synthesis of [MoO­(O_2_)­(pto)_2_] (**1**)

A mixture of Mo­(CO)_6_ (0.25 g, 0.95 mmol), Hpto (0.15 g, 0.95 mmol), and toluene (20 mL) was refluxed in a Schlenk tube for 30 min. Subsequently, hexane (40 mL) was added to promote product precipitation. A brown solid was isolated by filtration, washed with hexane (2 × 20 mL), and vacuum-dried for 2 h at rt. 1,2-Dichloroethane (20 mL) was added to the product followed by the dropwise addition of excess 5.0–6.0 M TBHP in decane (6 mL, ca. 33 mmol). The mixture was stirred for 24 h at 55 °C, during which time a yellow precipitate appeared. The solid was separated from the yellow solution by filtration, washed with hexane (2 × 10 mL), and vacuum-dried at rt for 2 h. Yield: 0.09 g, 20% (based on Mo­(CO)_6_). Single crystals of **1** suitable for X-ray diffraction were obtained by slow diffusion of diethyl ether into a solution of **1** in acetonitrile. Anal. Calcd for C_12_H_8_MoN_10_O_5_ (468.19): C, 30.78; H, 1.72; N, 29.92. Found: C, 30.91; H, 1.73; N, 29.77. Selected ATR FT-IR (cm^–1^): ν = 1622 (w), 1579 (w), 1531 (m), 1456 (sh), 1449 (s), 1385 (m), 1216 (sh), 1205 (s) (ν­(N–O)), 953 (vs) (ν­(Mo=O)), 917 (s) (ν­(O–O)), 841 (vs) (δ­(N–O)), 783 (vs), 599 (vs) (ν­(Mo­(O_2_)_2_)), 559 (vs) (ν­(Mo­(O_2_)_2_)), 527 (m). Selected Raman (cm^–1^): ν = 1623 (vs), 1579 (s), 1533 (s), 1450 (s), 1390 (w), 1228 (m), 1033 (m), 960 (m) (ν­(Mo=O)), 920 (m) (ν­(O–O)), 846 (m), 564 (m) (ν­(Mo­(O_2_)_2_)). ^1^H NMR (300.13 MHz, 25 °C, DMSO): δ = 8.77 (d, *J*_H–H_ = 6.5 Hz, 1H, H6), 8.54 (d, *J*_H–H_ = 7.8 Hz, 1H, H3), 8.18 (t, *J*_H–H_ = 7.8 Hz, 1H, H4), 7.93 (t, *J*_H–H_ = 6.5 Hz, 1H, H5) ppm.

### 2.2 Catalytic Tests

The catalytic reactions of Cy, methyl oleate (MO), methyl linoleate (ML), and dl-limonene (Lim) were carried out at 70 °C, using 1.8 mmol of substrate, solvent (1 mL), catalyst **1** (in an amount equivalent to 18 μmol of Mo), and TBHP or H_2_O_2_ as the oxidant. The reactions using TBHP (2.75 mmol for Cy; 3.85 mmol for biobased olefins) were carried out using borosilicate reactors with a 10 mL capacity fitted with a Teflon valve for sampling. Initially, a catalyst, solvent, substrate, and Teflon-lined magnetic stirring bar were added to the reactor, which was then immersed in an oil bath with the temperature set at 70 °C. After 10 min of stirring at 1000 rpm, preheated TBHP was added to the reactor, and timing of the reaction was started. The preheating operations were carried out to ensure isothermal catalytic conditions.

The reactions with H_2_O_2_ (1.37 mmol) were performed in tubular borosilicate batch reactors with pear-shaped bottoms, a sampling valve, and a capacity of ca. 12 mL. A catalyst, substrate, solvent, H_2_O_2_, and magnetic stirring bar were added to the reactor, which was then immersed in an oil bath heated to 70 °C, and stirring at 1000 rpm was started. Individual catalytic experiments were performed for each reaction time.

The reaction mixtures were analyzed using an Agilent 7820A GC fitted with a HP-5 capillary column (30 m × 0.320 mm × 0.25 μm; H_2_ as carrier gas) and an FID detector, for the quantification of reactants/products, based on calibrations. Undecane and methyl decanoate were used as internal standards for the substrates Cy/Lim and MO/ML, respectively. The substrate conversion was based on the initial number of moles of the limiting reactant (olefin for the **1**/TBHP systems, and oxidant for the **1**/H_2_O_2_ systems). The products were identified using Shimadzu GCMS-QP2010 Ultra equipment fitted with a Phenomenex capillary Zebron ZB5-MS column (ZB-5, 30 m × 0.25 μm × 0.25 mm; He as carrier gas) and commercial databases Wiley229 and NIST14.

After the Cy/TBHP reactions, the reaction mixtures were centrifuged (5000 rpm) to separate undissolved solids, which were washed with diethyl ether, acetone, or ethanol, dried under ambient conditions for 18 h, and then dried under reduced pressure (ca. 0.1 bar) at 60 °C for 1 h, leading to SP-TBHP-run*i* (*i* = 1, 2, 3). When the solids SP-TBHP-run1 and SP-TBHP-run2 were reused, the initial Cy:TBHP:Mo mass ratio was the same as that used in the first run. For the RISS catalytic system (**1**/Cy/H_2_O_2_), the self-precipitated solids of the consecutive batch runs were washed and dried as described above, leading to SP-H_2_O_2_-run*i*, where *i* = 1, 2, 3.

Contact tests were carried out for the **1**/TBHP system. Specifically, separate tests were carried out under similar conditions to those used for a normal catalytic run, but without olefin, for 5 h at 70 °C; then, the **1**/TBHP/solvent mixture was cooled to ambient temperature, and the undissolved solid catalyst was separated by centrifugation (5000 rpm), washed, and dried. The solid was subsequently used in a normal catalytic run. In parallel, the liquid phase (LP) of the contact test was filtered through a 0.2 μm PTFE membrane. The resultant solution was preheated at 70 °C for 10 min, after which preheated Cy was added to give an initial Cy concentration of 1.2 M (as used for a normal catalytic test). The resultant homogeneous mixture was stirred for 5 h at 70 °C, and Cy conversion was monitored by GC as described above. Soluble metal species (LP­(**1**/TBHP)) were isolated from the liquid phase of the contact test via the addition of 1:1 (v/v) diethyl ether and pentane to the solution and cooling to –4 °C.

## 3 Results and Discussion

### 3.1 Synthesis and Characterization

The title complex, [MoO­(O_2_)­(pto)_2_] (**1**), was obtained as a yellow solid directly from Mo­(CO)_6_ and the ligand Hpto in a one-pot synthesis that consisted of sequential thermal (toluene, reflux) and oxidative (TBHP, 55 °C) decarbonylation. The identity of **1** as a monooxo peroxo complex was confirmed by single-crystal XRD (described below). A search in the CCDC database^37^ revealed a limited number of examples of MoO­(O_2_)­L_2_-type complexes, which include those with L = 8-quinolinolate,^38^ oxazolinylphenolate,^39,40^ β-ketiminate,^41^ aryl hydroxamate,^42^ acylpyrazolonate,^43^ and iminophenolate ligands.^44–46^

Suitable crystals of **1** for XRD were obtained from a concentrated solution in MeCN. The complex crystallized in the centrosymmetric space group *P*2_1/*n*_, with the asymmetric unit composed of solely molybdenum complex that appeared highly disordered within the unit cell (Figure 1, Table 1). The Mo atom is hepta-coordinated by two pto^–^ organic residues, one peroxo group and one oxo group, with a coordination sphere, {MoO_5_N_2_}, that resembles a slightly distorted trigonal prism. Both pto^–^ residues are coordinated to the metal center in a bidentate fashion forming a *N*,*O*-chelate. This type of coordination leads to the nonplanar nature of the pto^–^ residues, both exhibiting a rotation between the aromatic and tetrazole ring (dihedral angles of 22.03 and 25.83°). This is in agreement with similar complexes containing Hpto molecules. While few structures have been reported (according to the CCDC database), all of them show a similar rotation between the aromatic and tetrazole rings (20.47–22.30° for the reported complexes).^47,48^ In fact, this rotation seems to be less pronounced when only one Hpto residue is included in the coordination sphere of a given complex, as reported recently by us for [MoO_2_Cl_2_(Hpto)]·THF (dihedral angle of 16.85°).^34^

**Figure 1:**
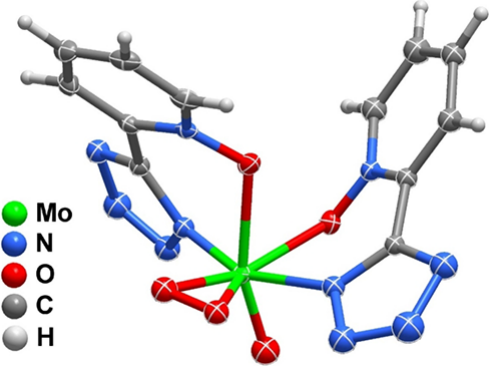
Representation of the molecular unit present in [MoO­(O_2_)­(pto)_2_] (**1**) showing all non-hydrogen atoms as displacement ellipsoids drawn at the 30% probability level and hydrogen atoms as small spheres with an arbitrary radius. For clarity, atomic labeling has been omitted, and only one position of the disordered molecular unit is represented.

**Table 1:** Crystal Data and Structure Refinement for [MoO­(O_2_)­(pto)_2_] (**1**)

formula	C_12_H_8_MoN_10_O_5_
formula weight	468.22
temperature/K	150(2)
crystal system	monoclinic
space group	*P*2_1_/*n*
*a*/Å	9.5045(4)
*b*/Å	11.0668(5)
*c*/Å	15.6878(8)
α/°	90.0
β/°	98.967(3)
γ/°	90.0
volume/Å^3^	1629.94(13)
*Z*	4
μ (Mo Kα)/mm^–1^	0.859
crystal type	yellow plate
crystal size/mm	0.29 × 0.28 × 0.06
θ range (°)	2.26–25.37
index ranges	–11 ≤ *h* ≤ 10, −13 ≤ *k* ≤ 13, −13 ≤ *l* ≤ 18
collected reflections	12324
independent reflections	2990 (*R*_int_ = 0.0458)
completeness to θ *=* 25.24°	99.7%
final *R* indices [*I* > 2σ(*I*)]	*R*_1_ = 0.0432, *wR*_2_ = 0.0867
final *R* indices (all data)	*R*_1_ = 0.0579, *wR*_2_ = 0.0927
largest diff. peak and hole/eÅ^–3^	0.586 and −0.482

The crystal packing of the individual [MoO­(O_2_)­(pto)_2_] complexes in **1** is solely mediated by very weak C–H···O supramolecular interactions with the complexes forming “pseudo square channels” along the [100] direction with the pto^–^ residues filling the channels (Figure 2).

**Figure 2:**
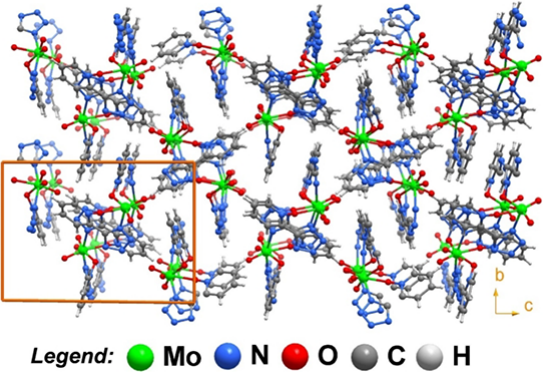
Schematic representation of the crystal packing of [MoO­(O_2_)­(pto)_2_] (**1**) viewed along the [100] direction.

The good phase purity of the bulk product **1** was confirmed by the close match between the experimental PXRD pattern and the simulated pattern calculated from the crystal structure data (Figure 3). In the solid-state, the ATR FT-IR spectrum of **1** shows characteristic strong absorption bands at 953 (Mo=O), 917 (O–O), and 599 and 559 cm^–1^ (Mo­(O_2_)) (Figure 4). Corresponding bands in the Raman spectrum are found at 564, 920, and 960 cm^–1^. A very strong band at 841 cm^–1^ in the IR spectrum of **1** (846 cm^–1^ with medium intensity in the Raman) is assigned to a ligand mode (N–O bending vibration) rather than ν­(O–O) since free Hpto displays a strong band at almost the same frequency (839 cm^–1^ in the IR, 841 cm^–1^ in the Raman). Several other ligand modes are observed in the 1000–1700 cm^–1^ range of the vibrational spectra of **1**. A strong IR band at 1205 cm^–1^ (with a shoulder at ca. 1216 cm^–1^) is assigned to a N–O stretching vibration and is an indicator for the involvement of the N–O group in coordination of the pto^–^ residue to the metal. A similar band was found at 1218 cm^–1^ for the compound [MoO_2_Cl_2_(Hpto)]·THF, which (as in **1**) possesses a bidentate *N*,*O*-coordinated ligand.^34^ The coordination of the pto^–^ residues to the metal center in **1** is further indicated by a shift of the band at 1612–1614 cm^–1^ for the free ligand Hpto (weak in IR, strong in the Raman), assigned to ν­(C=C) of the tetrazole ring, to 1622–1623 cm^–1^ for **1.**

**Figure 3:**
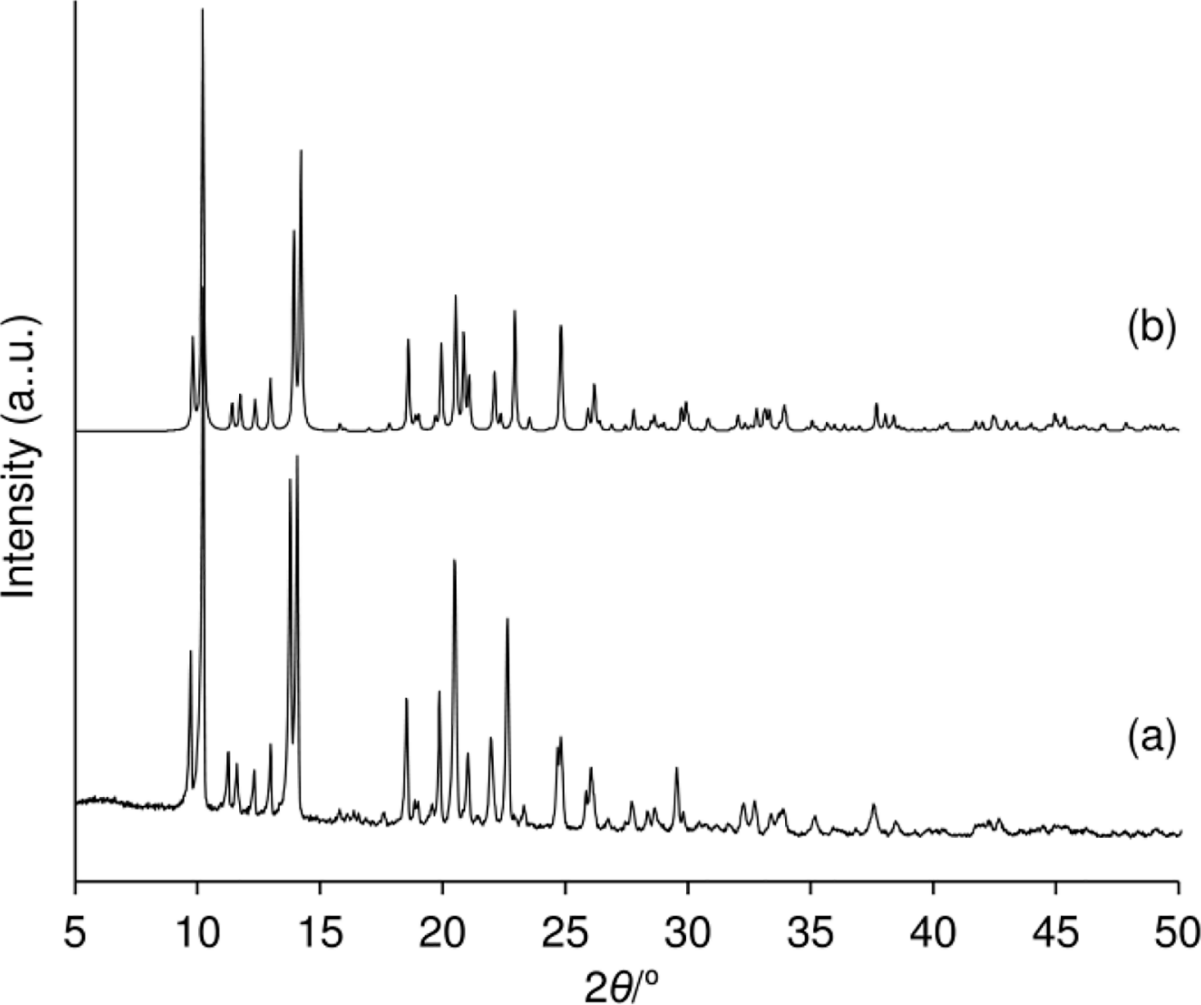
Experimental (a) and simulated (b) PXRD patterns of [MoO­(O_2_)­(pto)_2_] (**1**). The simulated pattern was calculated using the program Mercury^49^ and the corresponding crystal structure data for **1**.

**Figure 4:**
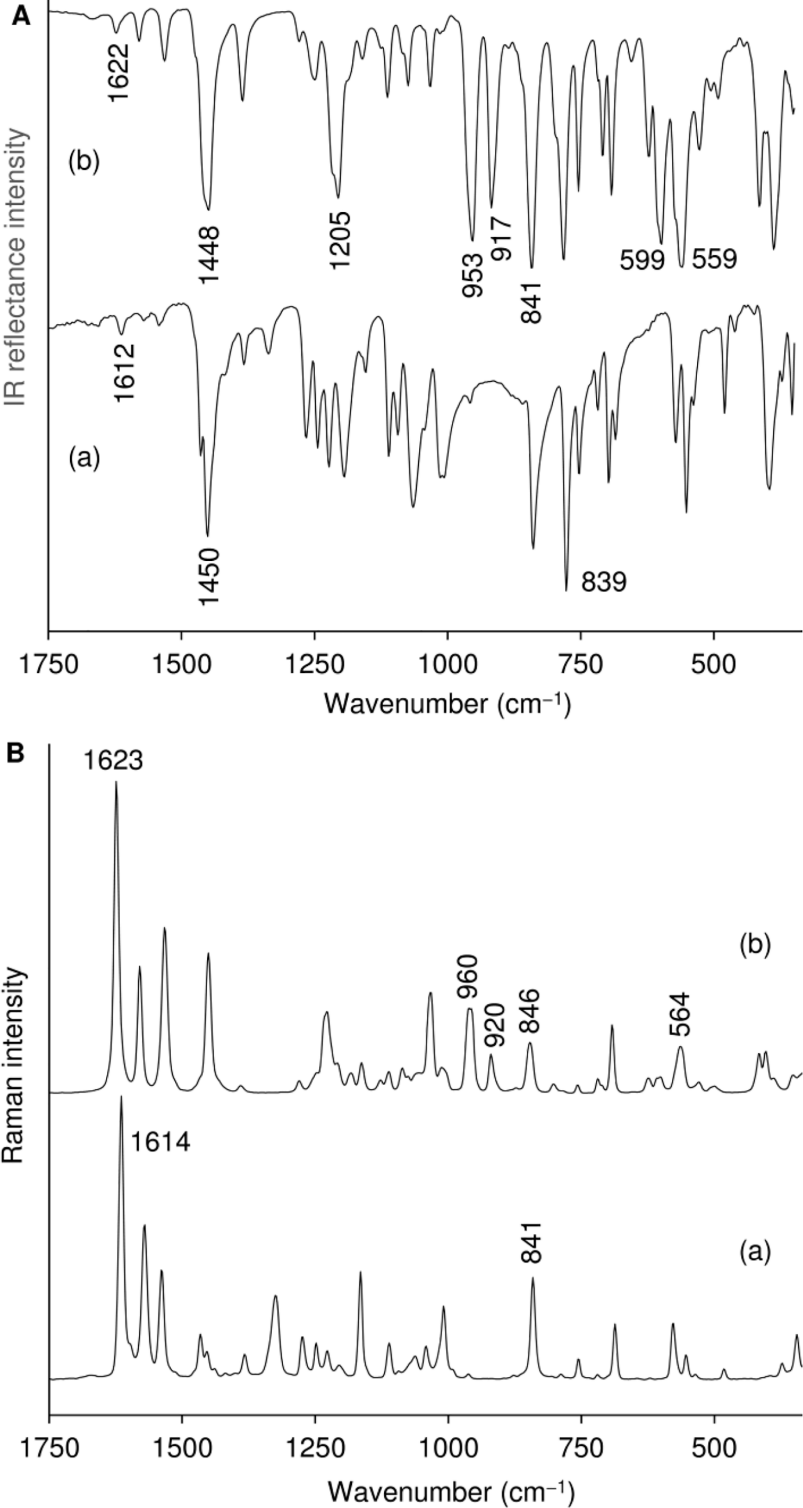
ATR FT-IR (A) and FT-Raman (B) spectra in the range 350–1750 cm^–1^ of (a) Hpto and (b) complex **1**.

### 3.2 Olefin Epoxidation with TBHP

#### 3.2.1 Model Reaction of *cis*-Cyclooctene and Solvent Effects

Compound **1** was first investigated for catalytic olefin epoxidation using *cis*-cyclooctene (Cy) as a model substrate, TBHP as oxidant, and different types of solvents, at 70 °C. In general, **1** led to 100% cyclooctene oxide (CyO) selectivity up to 100% Cy conversion within 4 h reaction (Figure 5). Without a catalyst and/or oxidant, olefin conversion was negligible. This is in line with literature studies of the mechanism of molybdenum-catalyzed epoxidation systems, which generally reveal that Lewis acid–base type reactions may occur between the molybdenum complex and the oxidant, leading to the formation of active oxidizing species.^50–55^ Different structures of oxidizing species (transition states) were reported and their reactivities may partly depend on the types of ligands and conditions.^25,53,55–57^ Thiel and co-workers reported that olefin epoxidation with monooxo-diperoxomolybdenum complexes (i.e., possessing the moiety {Mo­(O)­(O_2_)_2_}) and alkylhydroperoxide oxidants (ROOH) may begin with the activation of the oxidant by the starting metal complex, giving active oxidizing species, e.g., of the type {Mo­(O)­(O_2_)­(OOH)­(OOR)}; an O-atom from the −OOR ligand is then transferred to the olefin.^23,25^ Kühn and co-workers studied olefin epoxidation with TBHP in the presence of a monoooxo-monoperoxomolybdenum compound (i.e., possessing the moiety {Mo­(O)­(O_2_)}) and proposed that the oxidant activation leads to oxidizing species of the type {Mo­(O)­(OOH)­(OOR)}.^58^ Kühn’s epoxidation mechanism was distinct from that of Thiel in that it was suggested that an O-atom from the −OOH ligand (rather than from −OOR) is transferred to the olefin. A common feature of the above proposals is that an η^2^-peroxo ligand is opened in the process of oxidant activation or olefin epoxidation. In a distinct approach (i.e., keeping the η^2^-peroxo ligand intact), Calhorda and Poli and co-workers suggested that the interaction of ROOH with monooxo-monoperoxomolybdenum compounds ({Mo­(O)­(O_2_)}) may give oxidizing species with alkylperoxo and hydroxide ligands ({Mo­(O_2_)­(OH)­(OOR)}); then an O-atom from the −OOR ligand is transferred to the olefin, regenerating {Mo­(O)­(O_2_)}.^53,54^

**Figure 5:**
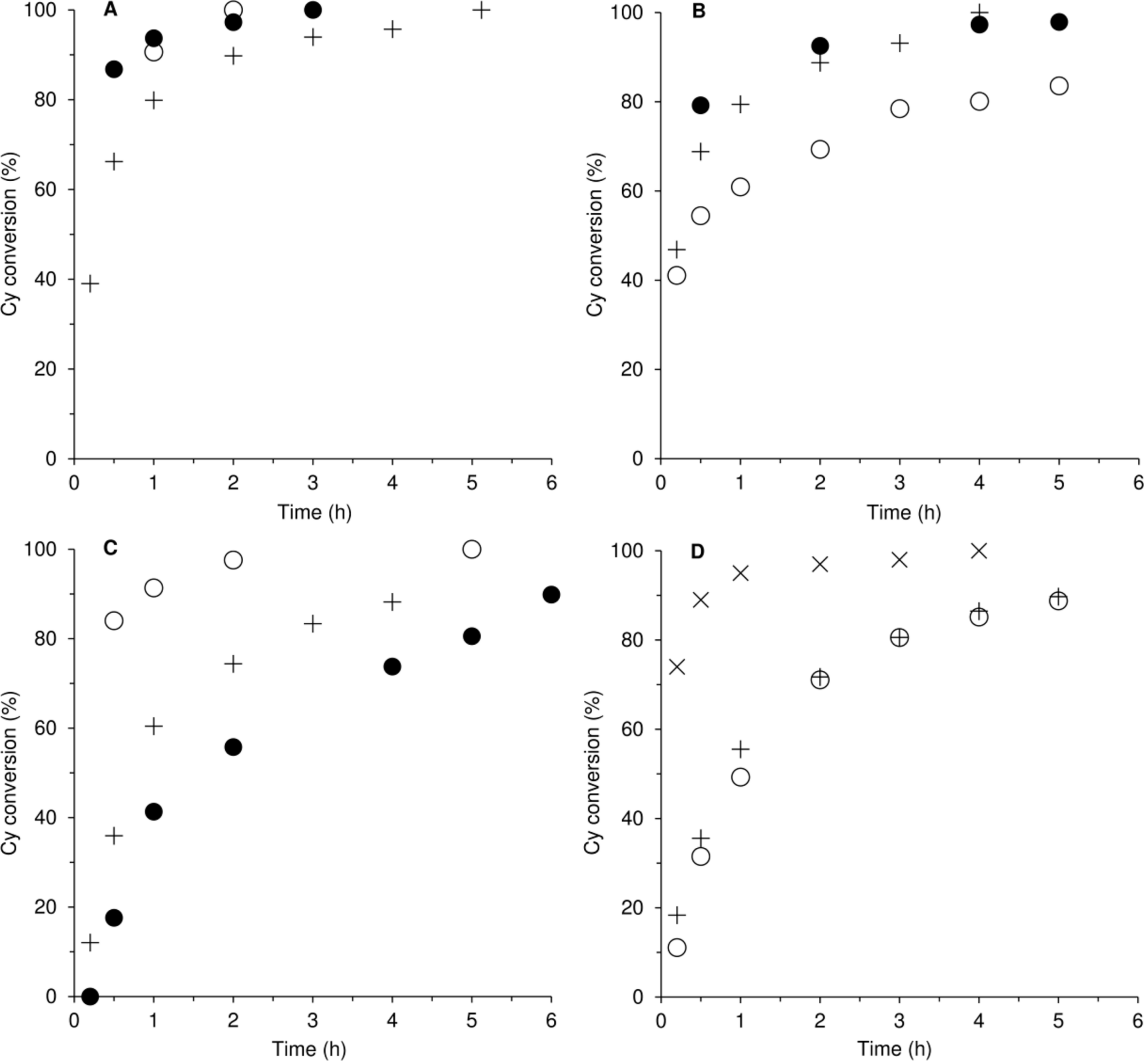
Kinetic profiles for the reaction of Cy with TBHP at 70 °C, in the presence of **1** (plus), the solid phase of the contact test (circle, closed), and the liquid phase of the contact test (circle, open), using α,α,α-trifluorotoluene (A), MeCN (B), ethyl acetate (C) and ethanol (D) as solvent (in the latter case, the catalyst was almost completely soluble). In part D, the kinetic profile for the reaction performed without the addition of a solvent is also shown (cross).

The influence of the type of solvent on the performance of the catalytic system **1**/TBHP was studied (Figure 5). Cy epoxidation was faster for α,α,α-trifluorotoluene (TFT) and acetonitrile as solvents (96–100% conversion at 4 h) than that for EtOH and ethyl acetate (EA) (86–88% conversion at 4 h). On the other hand, the kinetic profiles were roughly coincident for TFT/MeCN, and, on the other hand, for EA/EtOH. Coordinating solvents may compete with the oxidant for coordination to the molybdenum center,^50–55^ retarding the overall epoxidation reaction kinetics. However, there was no clear correlation between the catalytic results and the solvents’ coordinating properties (e.g., the kinetic curves are roughly coincident for (noncoordinating) TFT and (coordinating) MeCN), nor with the solvents’ dielectric constants^59^ or polarities. On the other hand, although the catalyst was almost completely soluble in EtOH, the reaction kinetics was slower than with TFT or MeCN, suggesting that catalyst solubility was not the sole factor responsible for the overall reaction kinetics. In summary, a complex interplay of factors may be responsible for the solvent effects. When the reaction was carried out without solvent, 100% conversion was reached within 4 h. The differences in catalytic results for the systems with solvent versus those without solvent may be partly due to differences in concentrations of the catalyst and reagents, influencing the reaction kinetics.

The system **1**/Cy/TBHP consisted of a biphasic solid–liquid mixture. Contact tests were carried out in which **1** was stirred in each solvent with TBHP (153 equiv) at 70 °C for 5 h, and the resultant solid–liquid mixture was then separated by centrifugation/filtration steps. The insoluble solid of the contact test was recovered in ca. 98.5% yield (by weight) relative to the starting amount. Afterward, Cy (for the filtrate) or Cy + TBHP + solvent (for the solid) was added, and the mixtures were stirred at 70 °C for 3–6 h, monitoring the evolution of the catalytic reactions by GC. The liquid phases of these tests led to 80–100% Cy conversion at 4 h, suggesting that **1**/TBHP is a homogeneous catalytic system. The solid phase of each contact test was yellow, similar to the color of **1**. When these solids were reused in a normal catalytic run, 74–100% Cy conversion was reached at 4 h, with 100% CyO selectivity (this test was not performed with EtOH as solvent because the catalyst almost completely dissolved). Soluble metal species (denoted as LP­(**1**/TBHP)) were isolated from the liquid phase of the contact test. These species exhibited an ATR FT-IR spectrum different from that of **1** (Figure 6i). Specifically, the spectrum displays characteristic bands for the Mo­(O_2_)_2_ moiety (ca. 530, 585, and 655 cm^–1^), the O–O groups (860 and 920 cm^–1^), the Mo=O groups (955 cm^–1^), and the organic ligand (778, 841, 1202, and 1219 cm^–1^). While the weak bands at 920 and 1202 cm^–1^ could be due to residual **1**, the other bands suggest that the soluble metal species may be primarily oxodiperoxomolybdenum-type compounds bearing the *N*,*O*-coordinated organic ligand. The material balance for the metal species of the contact test indicated that the liquid phase contained ca. 1.5 mol % of the initial amount of molybdenum. Accordingly, the turnover frequency is ca. 6000 mol mol_Mo_^–1^ h^–1^ (calculated for 1 h of the homogeneous catalytic reaction using the liquid phase of the contact test).

**Figure 6:**
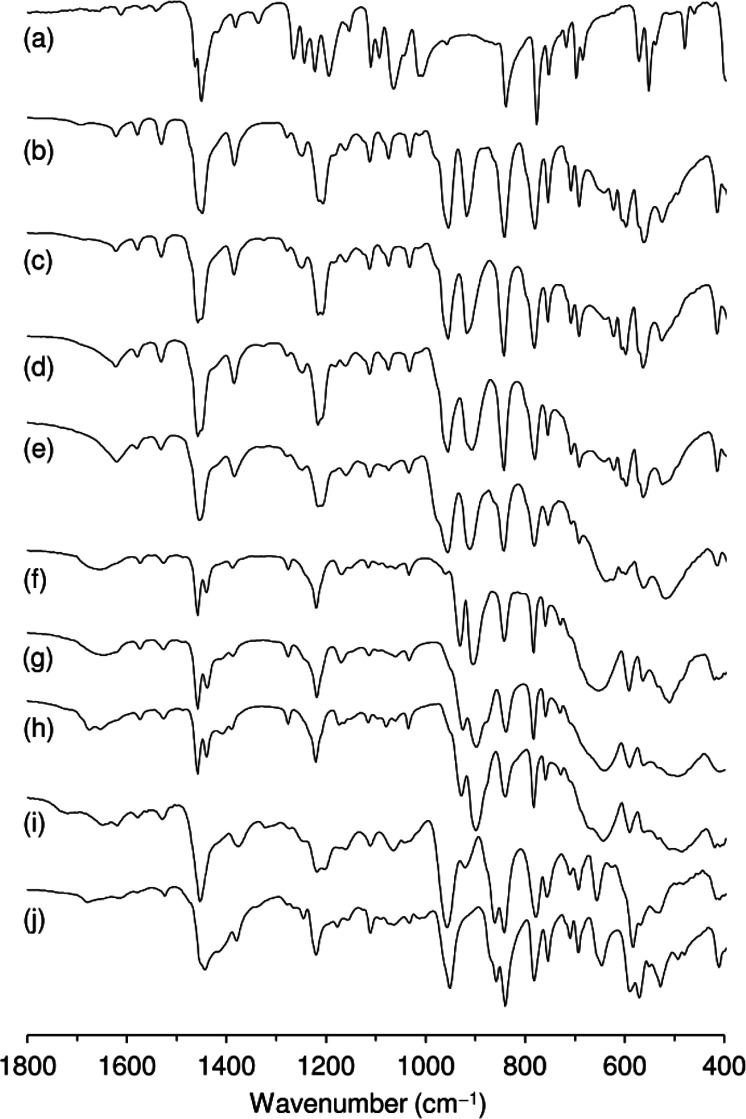
ATR FT-IR spectra of (a) Hpto, (b) **1**, (c, d) the solids recovered from batch runs with the reaction system Cy/TBHP/TFT (SP-TBHP-run1 (c), SP-TBHP-run3 (d)), (e) the solid recovered from the contact test of **1**/TBHP/TFT, (f–h) the solids recovered from the reaction system Cy/H_2_O_2_/MeCN after three consecutive batch runs (SP-H_2_O_2_-run1 (f), SP-H_2_O_2_-run2 (g), SP-H_2_O_2_-run3 (h)), (i) the soluble species LP­(**1**/TBHP), and (j) the soluble species LP­(**1**/H_2_O_2_).

The stability of **1** was further studied by reusing the recovered (solid phase) catalyst for the Cy/TBHP reaction at 70 °C (solvent = TFT). Catalyst **1** led to similar results for three consecutive batch runs (100% CyO selectivity at 100% conversion, 5 h), and the chemical structural features were preserved (based on ATR FT-IR spectroscopy, Figure 6). Overall, **1** seems to be relatively stable with TBHP, acting as a source of soluble metal species.

The catalytic reaction using aqueous TBHP (TBHPaq), in the presence of **1**, was slower than using TBHP in decane (MeCN, 70 °C): 42%/60%/81% Cy conversion at 1 h/4 h/24 h (100% CyO selectivity) for **1**/TBHPaq/MeCN, compared to 100% CyO yield within 4 h reaction for **1**/TBHP/MeCN. The poorer performance for TBHPaq may be at least partly due to the poor solubility of the olefin in water (possibly leading to mass transfer limitations), and on the other hand, water may compete with the reactants in the coordination to the metal center.

Table 2 compares the catalytic results for **1** with literature data for molybdenum compounds possessing tetrazole-type moieties, tested for the Cy/TBHP reaction at 70 °C.^32–34,60,61^ In four compounds, namely, [MoO_3_(*p*-trtzH)], [Mo_2_O_6_(*m*-trtzH)­(H_2_O)_2_], (H_2_ptz)_4_[SiMo_12_O_40_]·*n*H_2_O, and [*t*Bu-Hptz]_2_[Mo_6_O_19_], the tetrazole group is not coordinated to Mo atoms. With TFT or MeCN as solvent, **1** (entries 1 and 4) outperformed the silicododecamolybdate catalyst (entries 13 and 15) and molybdenum­(VI) oxide polymeric compounds (entries 6, 9, 11, and 12). On the other hand, using TFT as solvent, **1** was surpassed by complexes of the type [MoO_2_Cl_2_(L)] (entries 5, 8, and 17) and the hexamolybdate salt [*t*Bu-Hptz]_2_[Mo_6_O_19_] (entry 18), which all led to quantitative epoxide yield within 1 h.

**Table 2:** Comparison of the Catalytic Results for **1** with Literature Data for Molybdenum Compounds Possessing Tetrazole-Containing Organic Components, Tested for Epoxidation of Cy with TBHP

		reaction conditions^b^					
entry	catalyst^a^	solv.	TBHP:Cy	*t* (h)	conv.^c^ (%)	sel.^d^ (%)	ref.
1	**1**	TFT	1.5	4	96	100	this work
2				5	100	100	
3	**1**	MeCN	1.5	1	79	100	this work
4				4	100	100	
5	[MoO_2_Cl_2_(Hpto)]·THF	TFT	1.5	1	100	100	^34^
6	[MoO_3_(Hpto)]·H_2_O	TFT	1.5	4	82	100	^34^
7				24	100	100	
8	(H_2_ptz)[MoO_2_Cl_2_(ptz)]	TFT	1.5	1	100	100	^32^
9	[MoO_3_(Hptz)]	TFT	1.5	4	84	100	^32^
10				24	100	100	
11	[MoO_3_(*p*-trtzH)]	TFT	1.5	4	22	100	^60^
12	[Mo_2_O_6_(*m*-trtzH)(H_2_O)_2_]	TFT	1.5	4	14	100	^60^
13	(H_2_ptz)_4_[SiMo_12_O_40_]·*n*H_2_O	TFT	1.5	4	78	100	^61^
14				24	100	100	
15	(H_2_ptz)_4_[SiMo_12_O_40_]·*n*H_2_O	MeCN	1.5	4	44	100	^61^
16				24	90	97	
17	[MoO_2_Cl_2_(*t*Bu-ptz)]	TFT	1.5	0.2	100	100	^33^
18	[*t*Bu-Hptz]_2_[Mo_6_O_19_]	TFT	1.5	1	100	100	^33^

a Hptz = 5-(2-pyridyl)­tetrazole, *p*-trtzH = 5-[4-(1,2,4-triazol-4-ylphenyl)]-1*H*-tetrazole, *m*-trtzH = 5-[3-(1,2,4-triazol-4-ylphenyl)]-1*H*-tetrazole, *t*Bu-ptz = 2-*tert*-butyl-5-(2-pyridyl)-2*H*-tetrazole.

b 1 mol % molybdenum (based on olefin), 70 °C, solv. = solvent, initial TBHP:Cy molar ratio, *t* = reaction time.

c Cy conversion.

d CyO selectivity.

#### 3.2.2 Epoxidation of Biomass-Derived Olefins

Compound **1** was further explored for the catalytic epoxidation of biobased olefins, namely, the terpene dl-limonene (Lim) and the fatty acid methyl esters (FAMEs) methyl oleate (MO) and methyl linoleate (ML), with TBHP in TFT at 70 °C (Scheme 1). These bio-olefins are available from vegetable biomass or agricultural/industrial waste/residues and may be converted to renewable epoxides with broad application profiles. For example, Lim is the major component of citrus oils extracted from lemon, orange, grapefruit, and tangerine rinds and is also present in pine wood^62–64^ ; its epoxidation gives useful (di)­epoxides for manufacturing fragrances, cosmetics, pharmaceuticals, flavors, agrochemicals, biodegradable polymers, biomaterials, biofuels, paints, resins, coatings, and varnishes.^65–70^ The epoxidation of FAMEs obtained from vegetable oils such as canola oil gives (di)­epoxides with uses as PVC stabilizers, plasticizers, coatings, paints, varnishes, lubricants, soaps, inks, agrochemicals, biofuels, cosmetics, pharmaceuticals, and food additives.^71–77^

**Scheme 1:**
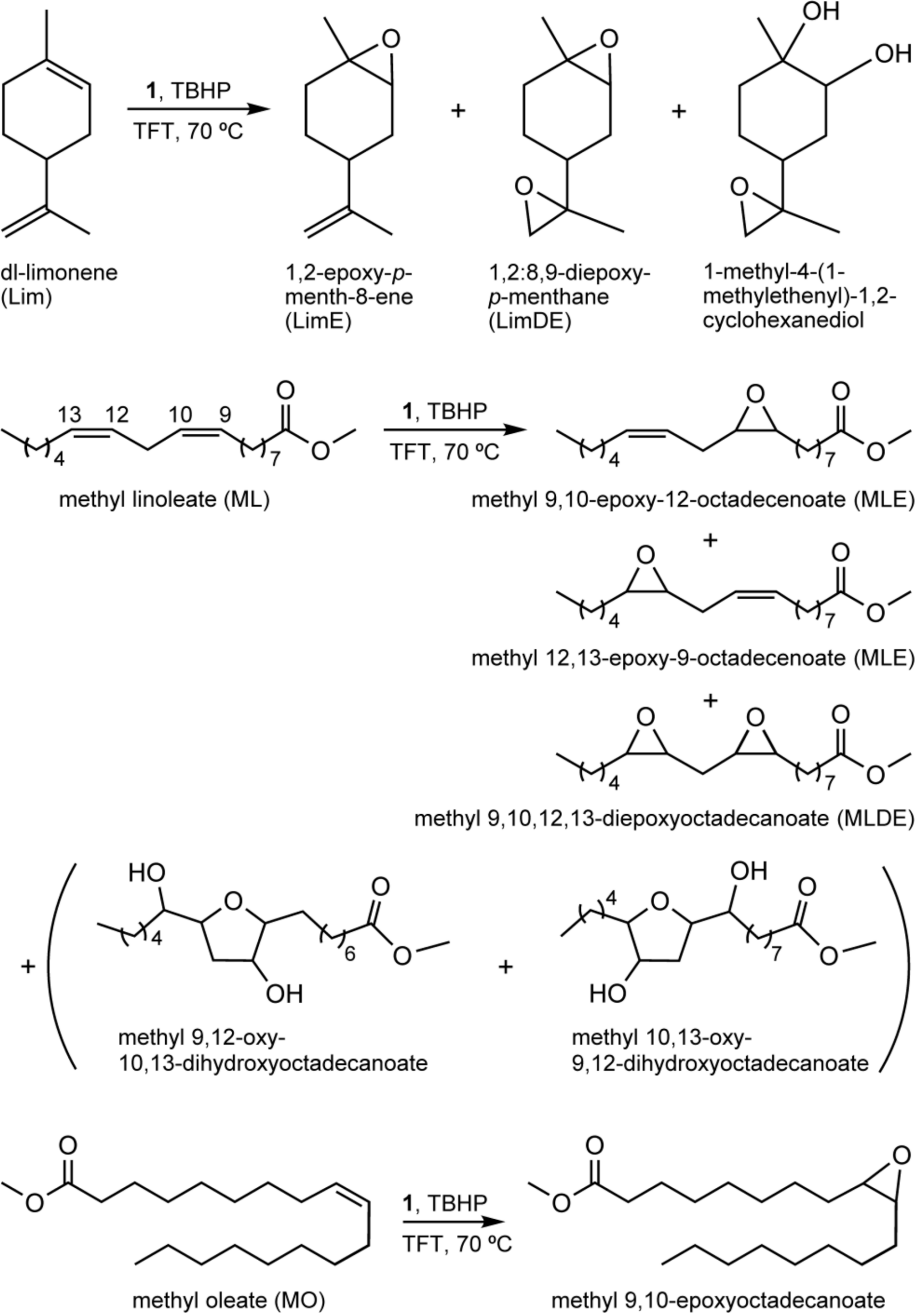
Epoxidation of Biomass-Derived Olefins to Useful Bio-products

Catalyst **1** promoted the epoxidation of the bio-olefins, giving up to 100% yield of the respective epoxide product at 24 h (Figure 7), whereas less than 5% conversion was reached for the three substrates without catalyst. The diene Lim was completely converted at 4 h, in the presence of **1**, giving mainly the monoepoxide 1,2-epoxy-*p*-menth-8-ene (LimE) in 68%/48% yield, and the diepoxide 1,2:8,9-diepoxy-*p*-menthane (LimDE) in 19%/21% yield, at 4 h/24 h (Figure 7). Other products included 1-methyl-4-(1-methylethenyl)-1,2-cyclohexanediol, which was formed in 9%/16% yield.

**Figure 7:**
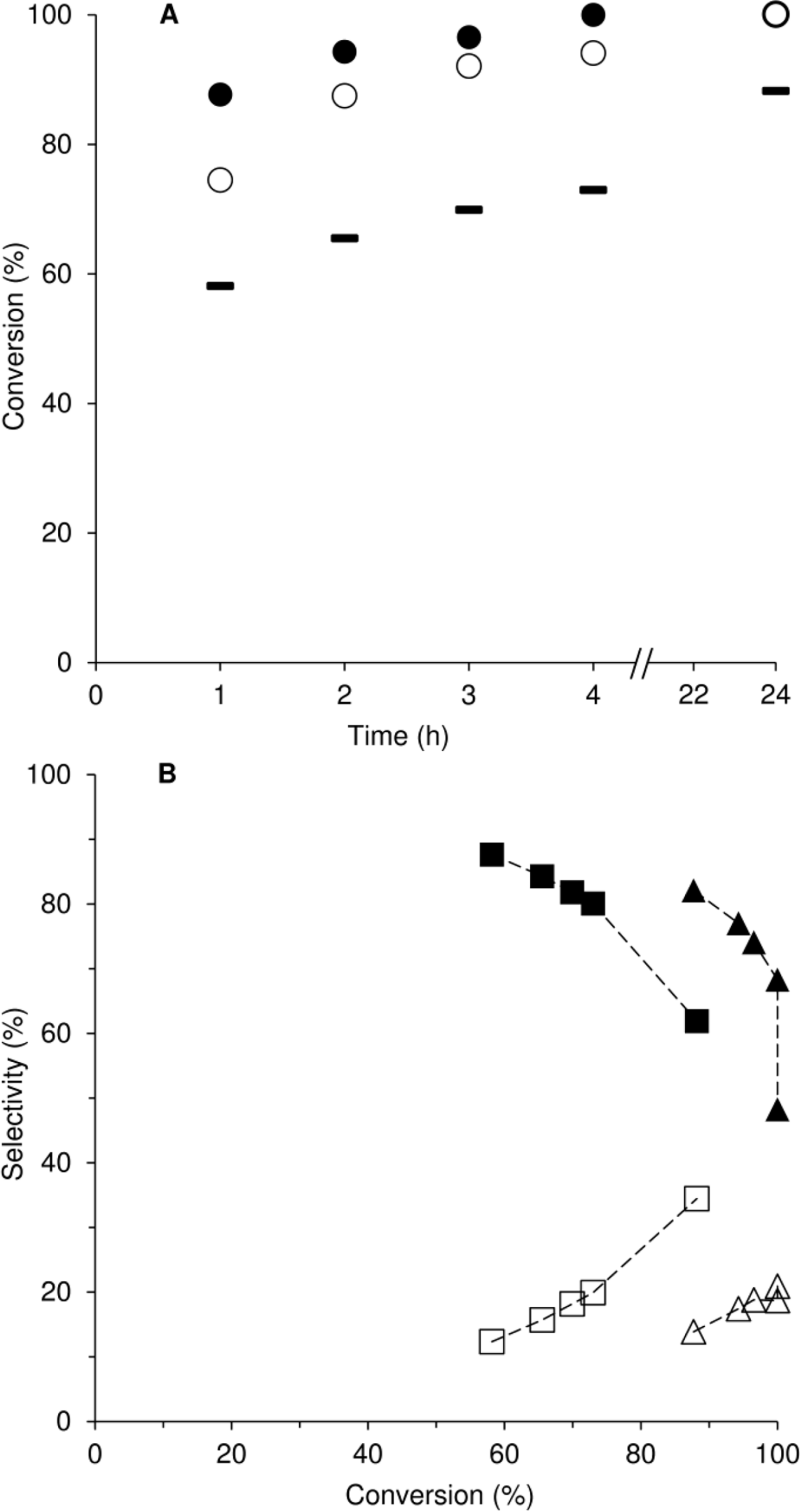
(A) Kinetic profiles for the catalytic reactions of the biobased olefins dl-limonene (circle, closed), methyl oleate (circle, open), and methyl linoleate (bar, closed) with TBHP, at 70 °C. (B) Dependency of selectivity on olefin conversion for the reaction of Lim (LimE (triangle, closed), LimDE (triangle, open)) and ML (MLE (box, closed), MLDE (box, open)).

The catalytic reaction of MO gave methyl 9,10-epoxyoctadecanoate with 100% selectivity at 94%/100% MO conversion, reached at 4/24 h (Figure 7). On the other hand, the catalytic reaction of ML gave mainly mono- and diepoxides with 100%/96% total selectivity at 73%/88% ML conversion, 4 h/24 h. The monoepoxide isomers, namely, methyl 9,10-epoxy-12-octadecenoate and methyl 12,13-epoxy-9-octadecenoate (MLE), were formed in total yields of 58%/55%, and the diepoxide methyl 9,10–12,13-diepoxyoctadecanoate (MLDE) was formed in 15%/30% yield, at 4 h/24 h. The other products of the ML reaction included methyl 9,12-oxy-10,13-dihydroxyoctadecanoate and methyl 10,13-oxy-9,12-dihydroxyoctadecanoate (3% yield at 24 h), which may be formed via intramolecular cyclization of diol intermediates.^78–81^ The kinetic profiles for the dienes Lim and ML show that as conversion increased, the monoepoxide selectivity decreased with the concomitant increase in diepoxide selectivity (Figure 7), suggesting that the monoepoxides were intermediates in the formation of the diepoxides.

Table 3 compares the catalytic results for **1** with literature data for molybdenum compounds possessing tetrazole-containing organic components, tested as catalysts for bio-olefin/TBHP conversion.^32–34,61^ On the whole, with the FAMEs as substrates, **1** performs either on a par or better than the four previously reported compounds (entries 11–19, 25–32), even though a higher initial TBHP:olefin molar ratio of 2.1 (vs 1.5 for **1**) was used for three of the four compounds, namely, [MoO_3_(Hpto)]·H_2_O, [MoO_3_(Hptz)] and [*t*Bu-Hptz]_2_[Mo_6_O_19_]. The only exception is the hexamolybdate, which performed slightly better in the epoxidation of MO, giving 97% epoxide yield after 3 h (entry 21) vs 94% at 4 h in the case of **1** (entry 12), but this difference may not be significant in the light of the different initial TBHP:MO ratios used. With Lim as the substrate, **1** performed better than (H_2_ptz)_4_[SiMo_12_O_40_]·*n*H_2_O, on par with [MoO_3_(Hptz)], and slightly worse than [MoO_3_(Hpto)]·H_2_O and [*t*Bu-Hptz]_2_[Mo_6_O_19_]. Although the results with **1** after 1 h of reaction were very good, with 88% conversion and 96% selectivity to epoxides (entry 1), the hexamolybdate led to complete conversion and 100% selectivity to epoxides after 30 min (entry 10).

**Table 3:** Comparison of the Catalytic Results for **1** with Literature Data for Molybdenum Compounds Possessing Tetrazole-Containing Organic Components, Tested for Epoxidation of Bio-olefins with TBHP

		react. conditions^a^				
entry	substrate/catalyst	TBHP:ole	*t* (h)	conv.^b^ (%)	sel.^c^ (%)	ref.
**Limonene**						
1	**1**	1.5	1	88	96	this work
2			4	100	87	
3			24	100	69	
4	(H_2_ptz)_4_[SiMo_12_O_40_]·*n*H_2_O	1.5	6	69	54	^61^
5			24	85	34	
6	[MoO_3_(Hpto)]·H_2_O	2.1	4	97	100	^34^
7			24	100	92	
8	[MoO_3_(Hptz)]	2.1	4	97	90	^32^
9			24	100	64	
10	[*t*Bu-Hptz]_2_[Mo_6_O_19_]	2.1	0.5	100	100	^33^
**Methyl oleate**						
11	**1**	1.5	1	75	100	this work
12			4	94	100	
13			24	100	100	
14	(H_2_ptz)_4_[SiMo_12_O_40_]·*n*H_2_O	1.5	6	88	100	^61^
15			24	100	100	
16	[MoO_3_(Hpto)]·H_2_O	2.1	4	79	100	^34^
17			24	96	100	
18	[MoO_3_(Hptz)]	2.1	4	76	100	^32^
19			24	100	100	
20	[*t*Bu-Hptz]_2_[Mo_6_O_19_]	2.1	0.5	73	100	^33^
21			3	97	100	
**Methyl linoleate**						
22	**1**	1.5	1	58	100	this work
23			4	73	100	
24			24	88	96	
25	(H_2_ptz)_4_[SiMo_12_O_40_]·*n*H_2_O	2.5	6	87	91	^61^
26			24	100	61	
27	[MoO_3_(Hpto)]·H_2_O	2.1	4	58	100	^34^
28			24	88	97	
29	[MoO_3_(Hptz)]	2.1	4	64	100	^32^
30			24	86	98	
31	[*t*Bu-Hptz]_2_[Mo_6_O_19_]	2.1	4	60	100	^33^
32			24	85	100	

a Reaction conditions: 1 mol % molybdenum (based on olefin), solvent = TFT, 70 °C, TBHP:ole = initial TBHP:olefin molar ratio, *t* = reaction time.

b Conversion.

c Selectivity to epoxides (in the case of Lim and ML, mono- plus diepoxides).

### 3.3 Catalytic Epoxidation Using H_2_O_2_

The performance of **1** was further explored using H_2_O_2_ as oxidant with different solvents, namely, MeCN, EtOH, and EA, at 70 °C (Figure 8). Catalyst **1** was effective for Cy/H_2_O_2_ epoxidation, leading to a 56–95% Cy conversion at 24 h. Without the catalyst, conversion was less than 5% (with MeCN as solvent). Conversion at 24 h increased in the order 56% (EA) < 92% (MeCN) ≅ 95% (EtOH). For the MeCN and EA solvent systems, CyO selectivity was 100%, whereas with EtOH, the epoxide was formed in 96% selectivity at 95% Cy conversion, 24 h. In the initial stage of the reaction, a biphasic solid–liquid system was obtained with MeCN and EtOH as solvents, and a triphasic liquid–liquid–solid system was obtained with EA as solvent. Hence, the poorer performance using EA may be at least partly due to mass transfer limitations. Overall, MeCN as solvent seemed a good compromise.

**Figure 8:**
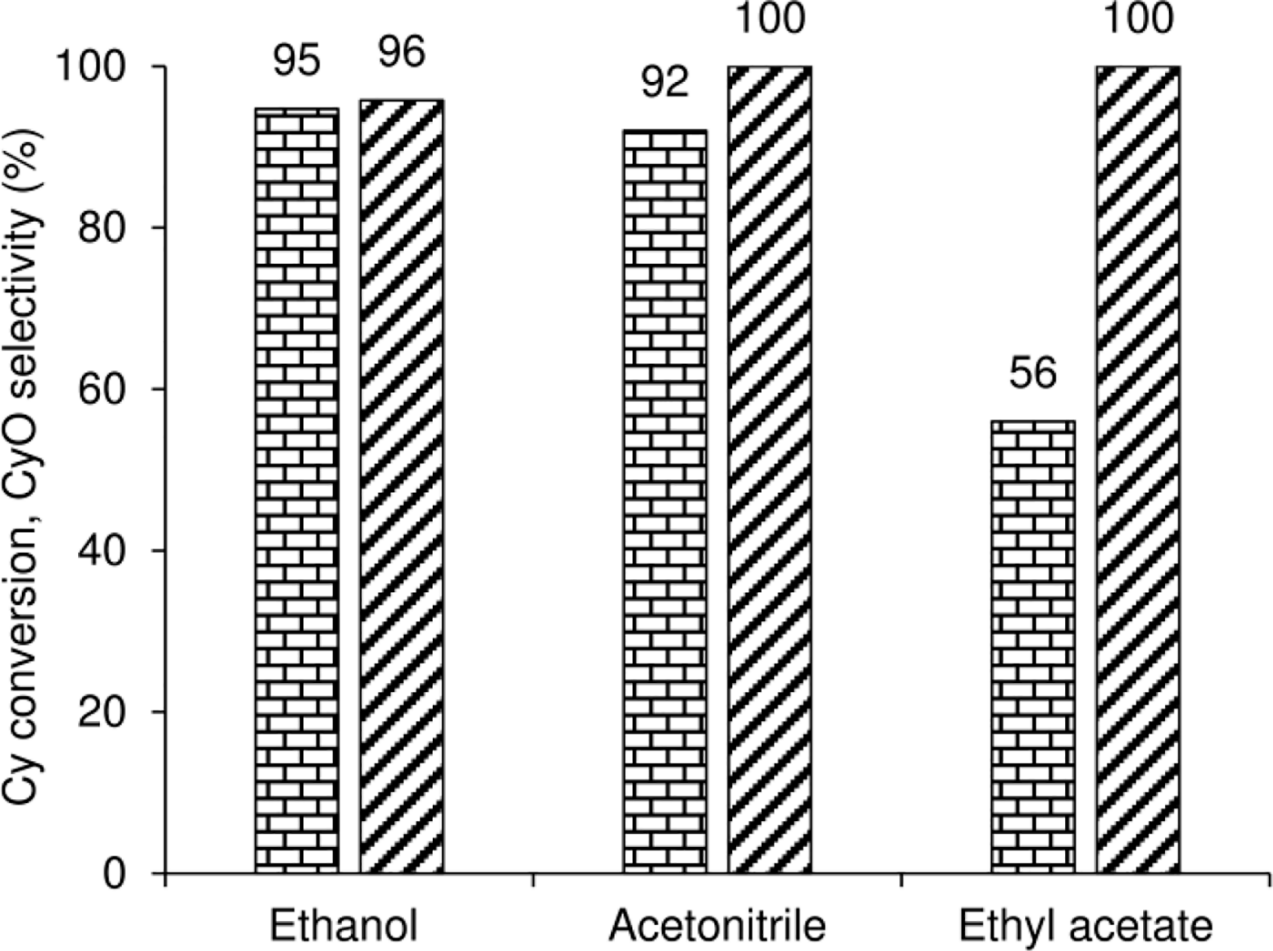
Cy conversion (bricks) and CyO selectivity (stripes) for the **1**/Cy/H_2_O_2_ reaction using different solvents at 70 °C, 24 h.

With MeCN as solvent, reaction-induced self-separating (RISS) behavior was observed. Thus, although the reaction mixture was initially biphasic solid–liquid, with time the yellow solid (**1**) was converted to soluble metal species, forming a homogeneous yellow liquid phase. At 24 h, the reaction mixture was again biphasic (but this time comprising a white solid and a colorless liquid phase); i.e., a solid catalyst self-precipitated upon consumption of the oxidant, allowing easy separation by filtration or centrifugation. The RISS solid was reused in consecutive batch runs performed at 70 °C using MeCN as solvent. Cy conversion at 24 h was 92%, 92%, and 90% in runs 1, 2, and 3, respectively, and CyO selectivity was always 100%, suggesting that the catalyst was relatively stable. The ATR FT-IR spectra of the solids recovered from each run (SP-H_2_O_2_-run*i*, *i* = 1, 2, 3) were similar to each other, but different from that of **1** (Figure 6), which correlates with the change in color from yellow for **1** to white for the recovered solids. The strong IR bands initially present at 953 (Mo=O) and 917 cm^–1^ (O–O) for **1** were replaced by bands centered at about 900 and 930 cm^–1^ for the recovered solids, while no change was observed regarding the positions of the ligand modes at 841 and 783 cm^–1^. Toward higher frequency, in the range 1150–1600 cm^–1^, the bands assigned to ligand modes changed slightly on going from **1** to the recovered solids, but nevertheless indicate the retention of a bidentate *N*,*O*-coordinated (H)­pto ligand. Specifically, the ν­(N–O) band at 1205 cm^–1^ for **1** (with a pronounced shoulder at *ca.* 1216 cm^–1^) was replaced by a single band at 1219 cm^–1^, similar to that exhibited by the compound [MoO_2_Cl_2_(Hpto)]·THF.^34^ The stronger band at 1449 cm^–1^ (with a shoulder at *ca.* 1456 cm^–1^) for **1** was replaced by two resolved bands at 1440 and 1458 cm^–1^, and the weaker bands at 1531 and 1579 cm^–1^ shifted to lower frequency by 4–5 cm^–1^. Although the ATR FT-IR spectra of the recovered solids indicate the presence of the coordinated organic ligand (possibly in the neutral form, Hpto), elemental (CHN) and ICP-OES (Mo) analyses of SP-H_2_O_2_-run1 indicated a (H)­pto:Mo molar ratio of 1 rather than 2 (as in **1**), with the composition MoO_3_(Hpto)·H_2_O being suggested.^82^ The structure of the RISS catalyst has not yet been completely identified. However, the presence of a polymeric molybdenum­(VI) oxide substructure is supported by the presence of very strong and broad IR bands centered at 511 and 652 cm^–1^, which may be due to stretching vibrations of bridging Mo–O–Mo or OMo_3_ units. It follows that the bands at 900 and 930 cm^–1^ can be assigned to the ν­(Mo=O) vibrations of *cis*-dioxo units.

The soluble metal species (LP­(**1**/H_2_O_2_)) of the RISS system **1**/H_2_O_2_/MeCN were isolated and characterized by ATR FT-IR spectroscopy (Figure 6j). In the region that contains bands assigned to Mo–O (terminal, bridging, or peroxo) stretching vibrations, the spectrum of LP­(**1**/H_2_O_2_) is clearly distinct from that of **1** or the recovered solids SP-H_2_O_2_-run*i*, and presents several spectral features of LP­(**1**/TBHP). First, in the region 880–980 cm^–1^, LP­(**1**/H_2_O_2_) only exhibits one strong band at 951 cm^–1^, which, if assigned to ν­(Mo=O), suggests the presence of a monooxo species like **1** but with a different structure because of the absence of the ν­(O–O) band at 917 cm^–1^. Moving to lower energy, in the region of 770–870 cm^–1^, LP­(**1**/H_2_O_2_) displays ligand modes at 782 and 840 cm^–1^, similar to those found for **1** and SP-H_2_O_2_-run*i*, and a new band with medium intensity at 859 cm^–1^. This band is assigned to ν­(O–O) and is a marker for the presence of a monooxo-diperoxo unit, MoO­(O_2_)_2_.^83,84^ The pattern of bands in the ν­(Mo­(O_2_)_2_) region is consistent with this assignment, with characteristic bands being observed at 529, 590, and 646 cm^–1^. Above 1000 cm^–1^, LP­(**1**/H_2_O_2_) displays a single ν­(N–O) band with a medium intensity at 1220 cm^–1^. Hence, the ATR FT-IR spectrum of LP­(**1**/H_2_O_2_) suggests that oxodiperoxomolybdenum complexes with coordinated (H)­pto moieties are the active metal species.

According to Galindo and co-workers,^85^ the mechanism of olefin epoxidation with H_2_O_2_ in the presence of monooxo-diperoxomolybdenum complexes may involve the direct interaction of the (nucleophilic) olefin with an (electrophilic) oxygen atom of a η^2^-coordinated peroxo ligand, forming a spirocyclic-type transition state, somewhat comparable to that proposed by Sharpless et al.^86^ Alternatively, depending on the coordination sphere and types of ligands in the starting molybdenum compound, it is possible that H_2_O_2_ activation occurs prior to olefin epoxidation via a Thiel-type mechanism; the oxidizing species may be of the type {Mo­(O)­(O_2_)­(OOH)_2_} (its formation involves η^2^-peroxo ring opening).^87^

Table 4 compares the catalytic results for **1**/H_2_O_2_ with literature data for molybdenum compounds possessing tetrazole-containing organic components, tested in the Cy/H_2_O_2_ reaction.^33,34,61,88^ Among these compounds, only **1**/H_2_O_2_ exhibited RISS behavior, and to the best of our knowledge RISS behavior has not been reported for any other catalyst bearing a tetrazole-based ligand. Compound **1** seemed to perform relatively well, leading to 100% CyO selectivity at 92% conversion (24 h), which is comparable with that found for [MoO_3_(*p*-trtzH)], [Mo_2_O_6_(*m*-trtzH)­(H_2_O)_2_] and (H_2_ptz)_4_[SiMo_12_O_40_]·*n*H_2_O (entries 3–5), and better than that found for [MoO_3_(Hpto)]·H_2_O and [*t*Bu-Hptz]_2_[Mo_6_O_19_] (entries 2 and 7).

**Table 4:** Comparison of the Catalytic Results for **1** with Literature Data for Molybdenum Compounds Possessing Tetrazole-Containing Organic Components, Tested for Epoxidation of Cy with H_2_O_2_

			reaction conditions^b^					
entry	catalyst	RISS?^a^	solv.	H_2_O_2_:Cy	*t* (h)	conv.^c^ (%)	sel.^d^ (%)	ref.
1	**1**	yes	MeCN	0.75	24	92	100	this work
2	[MoO_3_(Hpto)]·H_2_O	no	MeCN	1.5	24	81	100	^34^
3	[MoO_3_(*p*-trtzH)]	no	MeCN	1.5	24	93	100	^88^
4	[Mo_2_O_6_(*m*-trtzH)(H_2_O)_2_]	no	MeCN	1.5	24	96	100	^88^
5	(H_2_ptz)_4_[SiMo_12_O_40_]·*n*H_2_O	no	MeCN	1.5	24	91	100	^61^
6	(H_2_ptz)_4_[SiMo_12_O_40_]·*n*H_2_O	no	EA	1.5	24	96	91	^61^
7	[*t*Bu-Hptz]_2_[Mo_6_O_19_]	no	MeCN	1.5	4	58	100	^33^

a RISS = reaction induced-self-separating character.

b Reaction conditions: 1 mol % molybdenum (based on Cy), 70 °C, initial H_2_O_2_:Cy molar ratio, *t* = reaction time.

c Cy conversion, based on the limiting reactant.

d Selectivity to cyclooctene oxide.

## 4 Conclusions

In the present work, we have described the second example of a structurally characterized molybdenum­(VI) complex bearing the ligand 5-(2-pyridyl-1-oxide)­tetrazol­(e/ate). The first complex, [MoO_2_Cl_2_(Hpto)]·THF, has the ligand coordinated in a bidentate fashion in its neutral (protonated) form, while [MoO­(O_2_)­(pto)_2_] (**1**) has the ligand coordinated in a bidentate fashion in its anionic (deprotonated) form. The discovery of complex **1** is significant because it shows that the ligand Hpto can stabilize peroxomolybdenum­(VI) complexes in addition to dioxomolybdenum­(VI) complexes like [MoO_2_Cl_2_(Hpto)]. Hence, Mo^VI^–Hpto complexes are worth studying in the field of olefin epoxidation, where peroxo complexes are generally the catalytically active species. The catalytic results described here show that the activity of complex **1** is good in all the studied reactions, from the epoxidation of the model substrate Cy to the epoxidation of biobased olefins, and comparable to or better than related molybdenum compounds possessing tetrazole-containing organic components. The catalytic reaction was homogeneous using TBHP as the oxidant, whereas RISS behavior was observed using H_2_O_2_. In the latter case, **1** is converted via soluble active peroxo species to a hybrid molybdenum oxide that retains coordinated Hpto ligands and can be repeatedly reused as a RISS catalyst for the epoxidation reaction. The results encourage further work to explore the potential of Hpto as a ligand to enable RISS behavior in metal-catalyzed olefin epoxidation.

## Supplementary Material





## Notes

Crystallographic information for compound **1** (CIF)
Sources and purities of reagents, solvents and substrates, specifications of instrumentation used for characterization, and experimental details for the single-crystal X-ray diffraction studies (PDF)

## Notes

Deposition Number 2449047 contains the supplementary crystallographic data for this paper. These data can be obtained free of charge via the joint Cambridge Crystallographic Data Centre (CCDC) and Fachinformationszentrum Karlsruhe Access Structures service.
