# Supplementary material for: Oxidant-Dependent Switch of a Molybdenum(VI) Tetrazolate Complex from a Homogeneous to a Self-Separating Catalyst for Olefin Epoxidation
Source: Ind Eng Chem Res. 2025 Aug 18;64(34):16589–601. doi: 10.1021/acs.iecr.5c01997 (PMC12821073; doi:10.1021/acs.iecr.5c01997)
Supplement: Supplementary file 2 [file ie5c01997_si_002.pdf]

Supporting Information

Oxidant-dependent switch of a molybdenum(VI) tetrazolate complex from a homogeneous to a self-separating catalyst for olefin epoxidation

Martinique S. Nunes, Diana M. Gomes, Patrícia Neves, Ana C. Gomes, Ricardo F. Mendes, Filipe A. Almeida Paz, Isabel S. Gonçalves, Anabela A. Valente,* Martyn Pillinger*

CICECO – Aveiro Institute of Materials, Department of Chemistry, University of Aveiro, Campus Universitário de Santiago, 3810-193 Aveiro, Portugal

* Corresponding authors
atav@ua.pt (A.A.V.), mpillinger@ua.pt (M.P.)

1. EXPERIMENTAL DETAILS

1.1. Materials. Molybdenum hexacarbonyl, potassium carbonate (99%), 5.0-6.0 M *tert*-butyl hydroperoxide in decane, 30 wt.% H_2O_2, pentane (≥ 99.5%), hexane (99%, Carlo Erba), 1,2-dichloroethane (≥ 99.0%), acetonitrile (99.9%, Panreac), ethyl acetate (99.9%), toluene (≥ 99.7%), anhydrous α,α,α-trifluorotoluene (≥ 99%), anhydrous ethanol (99.9%, Carlo Erba), acetone (99.5%), diethyl ether (99.8%), *cis*-cyclooctene (95%, Alfa Aesar), methyl oleate (99%), methyl linoleate (95%, Alfa Aesar), dl-limonene (> 95%, Merck), methyl decanoate (99%) and undecane (> 99%) were purchased from Sigma-Aldrich (unless indicated otherwise) and used as received. FOMBLIN Y perfluoropolyether vacuum oil (LVAC 140/13) was obtained from Sigma-Aldrich. The ligand Hpto was prepared as described previously.[1]

1.2. Instrumentation. Microanalyses (CHN) were performed with a Truspec Micro CHNS 630-200-200 instrument. ICP-OES analyses for Mo (with a detection limit of *ca.* 20 μg dm^{-3} and an experimental error of 5%) were performed at the Central Analysis Laboratory, University of Aveiro, using a Horiba JobinYvon Activa M spectrometer. Powder X-ray diffraction (PXRD) data were collected at room temperature (rt) on a Malvern Panalytical Empyrean diffractometer fitted with a spinning flat plate sample holder and a PIXcel 1D detector set at 240 nm from the sample, in a Bragg-Brentano *para*-focusing optics configuration. Cu-$K\alpha_{1,2}$ X-radiation (λ_1 = 1.5406 Å) was used, with the X-ray tube operating at 45 kV and 40 mA. Samples were step-scanned in continuous mode from 5 to 70° (2θ) with step sizes of 0.026°, a counting time of 99 s per step, and automatic data acquisition (X'Pert Data Collector software v4.2). Attenuated total reflectance (ATR) FT-IR spectra were obtained in the spectral range of 350 to 4000 cm^{-1} using a Bruker Tensor 27 spectrometer fitted with a Specac Golden Gate Mk II ATR accessory having a diamond top plate and KRS-5 focusing lenses (resolution 4 cm^{-1}, 256 scans). Raman spectra were recorded on a Bruker MULTIRAM instrument fitted with a Nd:YAG laser, with an excitation wavelength of 1064 nm, 1000 scans, and a resolution of 4 cm^{-1}. ^1H NMR spectra were recorded on a Bruker Avance 300 spectrometer.

1.3. Single-crystal X-ray diffraction studies. Single-crystals of compound $[MoO(O_2)(pto)_2]$ (**1**) were manually selected from the crystallization vial and immersed in highly viscous FOMBLIN Y perfluoropolyether vacuum oil to prevent degradation caused by the evaporation of the solvent.[2] Crystals were mounted on either Hampton Research CryoLoops or MiTeGen MicroLoops, typically with the help of a Stemi 2000 stereomicroscope equipped with Carl Zeiss lenses.

Crystal data were collected at 150(2) K on a Bruker X8 Kappa APEX II CCD area-detector diffractometer (Mo Kα graphite-monochromated radiation, λ = 0.71073 Å) controlled by the APEX3 software package[3] and equipped with an Oxford Cryosystems 700 Series cryostream

cooler monitored remotely using Cryopad.[4] Diffraction images were processed using SAINT+,[5] and data were corrected for absorption by the multiscan semi-empirical method implemented in SADABS 2016/2.[6]

The structure was solved using the algorithm implemented in SHELXT-2014/5,[7] which allowed the immediate location of almost all the heaviest atoms composing the molecular unit. The remaining missing and misplaced non-hydrogen atoms were located from difference Fourier maps calculated from successive full-matrix least-squares refinement cycles on F^2 using the latest SHELXL from the 2018/3 release.[8] All structural refinements were performed using the graphical interface ShelXle.[9] The molecular unit present in **1** was found to be severely affected by positional disorder and several models were used to ultimately refine the presented crystal structure. In short, some portions of the molecular unit could be accommodated in different locations inside the unit cell while maintaining the same crystal features.

Hydrogen atoms bound to carbon were placed at their idealised positions using HFIX instructions in SHELXL: *43* for aromatic carbon atoms. These hydrogen atoms were included in subsequent refinement cycles with isotropic thermal displacement parameters (U_{iso}) fixed at 1.2 × U_{eq} of the parent carbon atoms. The last difference Fourier map synthesis showed the highest peak (0.586 eÅ^{-3}) and the deepest hole (−0.482 eÅ^{-3}) located at 0.73 and 0.83 Å from N4 and Mo2, respectively.

All structural drawings were created using Crystal Impact Diamond.[10] Crystallographic data (including structure factors) for the crystal structure of compound **1** have been deposited with the Cambridge Crystallographic Data Centre (CCDC) as supplementary publication no. CCDC-2449047. These data can be obtained free of charge from the CCDC via www.ccdc.cam.ac.uk/structures.

References

(1) M. S. Nunes, D. M. Gomes, A. C. Gomes, P. Neves, R. F. Mendes, F. A. A. Paz, A. D. Lopes, M. Pillinger, A. A. Valente, I. S. Gonçalves, A Molybdenum(VI) complex of 5-(2-pyridyl-1-oxide)tetrazole: Synthesis, structure, and transformation into a MoO_3-based hybrid catalyst for the epoxidation of bio-olefins, *Catalysts* **2023**, *13*, 565. https://doi.org/10.3390/catal13030565

(2) T. Kottke, D. Stalke, Crystal handling at low temperatures, *J. Appl. Crystallogr.* **1993**, *26*, 615-619. https://doi.org/10.1107/S0021889893002018

(3) APEX3 Data Collection Software, Version 2016.9-0, Bruker AXS Inc., Delft, The Netherlands, 2005-2016.

(4) Cryopad, Remote Monitoring and Control, Version 1.451, Oxford Cryosystems, Oxford, United Kingdom, 2006.

(5) SAINT+ Data Integration Engine, Version 8.37a, Bruker AXS Inc., Madison, WI, USA, 1997-2015.

(6) L. Krause, R. Herbst-Irmer, G. M. Sheldrick, D. Stalke, Comparison of Silver and Molybdenum Microfocus X-Ray Sources for Single-Crystal Structure Determination, *J. Appl. Crystallogr.* **2015**, *48*, 3-10. https://doi.org/10.1107/S1600576714022985
(7) G. M. Sheldrick, *SHELXT* - Integrated Space-Group and Crystal-Structure Determination, *Acta Crystallogr. A Found. Adv.* **2015**, *71*, 3-8. https://doi.org/10.1107/S2053273314026370
(8) G. M. Sheldrick, Crystal Structure Refinement with *SHELXL*, *Acta Crystallogr. C Struct. Chem.* **2015**, *71*, 3-8. https://doi.org/10.1107/S2053229614024218
(9) C. B. Hübschle, G. M. Sheldrick, B. Dittrich, *ShelXle*: A Qt Graphical User Interface for *SHELXL*, *J. Appl. Crystallogr.* **2011**, *44*, 1281-1284. https://doi.org/10.1107/S0021889811043202
(10) H. Putz, K. Brandenburg, Diamond—Crystal and Molecular Structure Visualization, Version 3.2f, Crystal Impact GbR, Bonn, Germany, 2010.
